# Computational Reconstruction of the Transcription Factor Regulatory Network Induced by Auxin in *Arabidopsis thaliana* L.

**DOI:** 10.3390/plants13141905

**Published:** 2024-07-10

**Authors:** Nadya A. Omelyanchuk, Viktoriya V. Lavrekha, Anton G. Bogomolov, Vladislav A. Dolgikh, Aleksandra D. Sidorenko, Elena V. Zemlyanskaya

**Affiliations:** 1 Department of Systems Biology, Institute of Cytology and Genetics SB RAS, 630090 Novosibirsk, Russia; nadya@bionet.nsc.ru (N.A.O.); vvl@bionet.nsc.ru (V.V.L.); mantis_anton@bionet.nsc.ru (A.G.B.); dolgikh@bionet.nsc.ru (V.A.D.); a.sidorenko1@g.nsu.ru (A.D.S.); 2 Department of Natural Sciences, Novosibirsk State University, 630090 Novosibirsk, Russia

**Keywords:** microarray, transcriptome, auxin, transcription factor, regulatory network

## Abstract

In plant hormone signaling, transcription factor regulatory networks (TFRNs), which link the master transcription factors to the biological processes under their control, remain insufficiently characterized despite their crucial function. Here, we identify a TFRN involved in the response to the key plant hormone auxin and define its impact on auxin-driven biological processes. To reconstruct the TFRN, we developed a three-step procedure, which is based on the integrated analysis of differentially expressed gene lists and a representative collection of transcription factor binding profiles. Its implementation is available as a part of the CisCross web server. With the new method, we distinguished two transcription factor subnetworks. The first operates before auxin treatment and is switched off upon hormone application, the second is switched on by the hormone. Moreover, we characterized the functioning of the auxin-regulated TFRN in control of chlorophyll and lignin biosynthesis, abscisic acid signaling, and ribosome biogenesis.

## 1. Introduction

Reconstruction of a gene regulatory network involved in the response to a certain stimulus (such as a hormone, an environmental cue, a mutation and others) using the whole genome data provides not only insights into the regulatory mechanisms but also may help to find a way to modify the effect of the stimulus [1,2,3,4,5]. A signaling cascade initiated by an external or internal stimulus usually results in an activation of transcription factors (TFs), which bind to DNA and regulate transcription of target genes [6,7]. These TFs affect transcription of genes, encoding other TFs, which can also have TF encoding genes as their targets. Altogether, they constitute a so-called transcription factor regulatory network (TFRN), which governs various biological processes. The increasing number of whole genome data on TF binding and transcriptome profiling necessitates making TFRN reconstruction a routine procedure accessible to any biologist. The predicted TFRNs allow determination of hierarchy in their structure and identification of hubs (highly interconnected nodes, which assimilate numerous signals from other TFs and form an output important for TFRN functioning) [4,8]. Development of methods for computational inference of TFRNs enables systematic study of the mechanisms underlying transcriptional responses to various stimuli [9,10,11].

Auxin is a primary regulator of plant development [12]. It alters gene expression by activation of the TFs belonging to the AUXIN RESPONSE FACTOR (ARF) family. Namely, auxin binding to nuclear TRANSPORT INHIBITOR RESPONSE 1 (TIR1) and AUXIN SIGNALING F-BOX (AFB) receptors promotes degradation of ARF repressors AUXIN/INDOLE-3-ACETIC ACID (AUX/IAA), thereby relieving ARFs, the master TFs of auxin response. In contrast to the auxin signaling pathway, which is extensively studied, the TFs downstream of ARFs, are not well characterized. ChIP-seq data on ARF3,5,6,7,10 [13,14,15], DNA affinity purification and sequencing (DAP-seq) data on ARF5 and ARF2 in *Arabidopsis thaliana* [16] and ARFs in maize [17] provided the list of putative direct ARF targets. At the same time, the analysis of auxin-induced transcriptome data predicted a set of auxin-sensitive TFs [14,18,19]. In fine-scale experiments, a number of auxin-sensitive TF-encoding genes were characterized in terms of their involvement in regulation of several auxin-driven biological processes. These include, for example, *LATERAL ORGAN BOUNDARIES-DOMAIN 16* (*LBD16*), *LBD18*, *LBD29*, *LBD33*, *GATA BINDING PROTEIN 23* (*GATA23*), *TARGET OF MONOPTEROS 5* (*TMO5*), *TMO6*, *DNA BINDING WITH ONE FINGER 5.8* (*DOF5.8*), and *ADENOVIRUS E2 PROMOTER BINDING FACTOR 2* (*E2F2*) [20,21,22,23,24]. Of them, *TMO5*, *TMO6*, *DOF5.8*, *LBD16* and *LBD29* are direct (primary) ARF targets [20,23,24], whereas *E2F2* and *GATA23* are indirect (secondary) ARF targets, their expression in response to auxin is directly regulated by LBD18/LBD33 and LBD16, respectively, [21,25]. Thus, the roles of the vast majority of auxin-sensitive TF-coding genes and the regulatory relations between them are poorly understood. This gap in our knowledge is important, since the primary targets of a TF constitute only 20–30% of differentially expressed genes (DEGs) in response to changes in its activity [26,27]. At least in *A. thaliana* and maize, this estimate was shown by comparing the lists of genes bound by TFs in ChIP-seq experiments with the lists of DEGs in their loss- and/or gain-of-function mutants. Previous attempts to reconstruct TF-centered auxin-responsive gene regulatory networks mostly focused on primary ARF targets in *A. thaliana* [28,29,30] and maize [31]. However, the existence of a large fraction of non-primary targets of master TFs raises the questions of how they are organized and what their functions are.

Herein, we identified TFs acting upstream auxin-induced DEGs and the regulatory relationships between them in *A. thaliana*. To reconstruct the TFRN affected by auxin, we developed a three-step procedure, which is based on the integrated analysis of DEG lists and a representative collection of TF binding profiles. As a result, we identified transcriptional subnetworks, which operate before auxin treatment and are switched off upon auxin application, and those that are switched on by auxin. Moreover, we illustrated the functioning of the TFRN in regulation of chlorophyll and lignin biosynthesis, ABA signaling and ribosome biogenesis.

## 2. Results

### 2.1. FindTFnet Implements a New Approach for Computational Reconstruction of TFRNs

**Figure 1:**
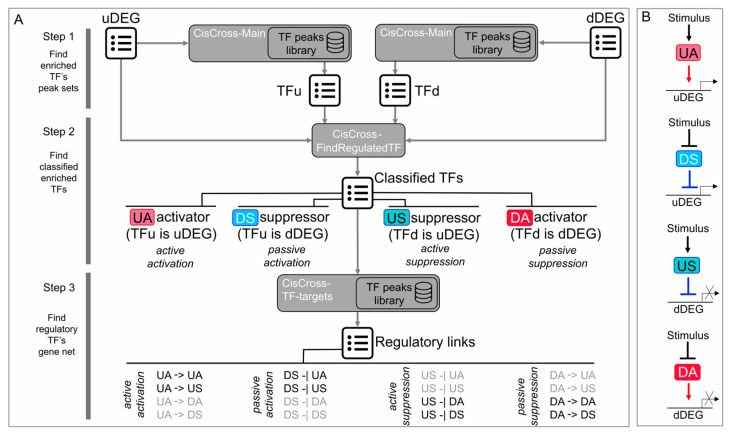
Reconstruction of induced TFRNs with FindTFnet. (**A**) A three-step procedure to infer “TF regulator–TF target” pairs. Step 1 searches for TFs, whose binding loci are enriched in 5′ regulatory regions of DEGs, employing *A. thaliana* DAP-seq TF peaks library. Step 2 classifies enriched TFs into four TF classes extracted for further analysis (the detailed description is in the text). Step 3 reconstructs “TF regulator–TF target” pairs. TF1 and TF2 are connected with a link directed from TF1 (regulator) to TF2 (target) if there is a TF1 binding peak in the 5′ regulatory region of the TF2 coding gene. The callout depicts all possible “TF regulator–TF target” pairs, which could be hypothetically reconstructed between distinct TF classes. The edge type (visualized as an arrow for activation and a bar-headed line for inhibition) is assigned according to the predicted function of the TF regulator (activator or suppressor). The regulatory links depicted in black are mechanistically meaningful, the ones depicted in light gray are inconsistent. (**B**) Classification of TFs involved in response to a factor. Red arrows and blue bar-headed lines depict the inferred regulations. uDEGs, upregulated DEGs; dDEGs, downregulated DEGs; TFu, TF enriched in 5′ regulatory regions of uDEGs; TFd, TF enriched in 5′ regulatory regions of dDEGs; DA, Downregulated Activator; DS, Downregulated Suppressor; US, Upregulated Suppressor; UA, Upregulated Activator.

We developed a three-step procedure (FindTFnet) for a large-scale reconstruction of TFRNs affected by certain stimuli based on the analysis of DEG lists (Figure 1A). First, to predict the upstream regulators of the sensitive genes, we search for TFs, whose binding loci are enriched in 5′ regulatory regions of downregulated DEGs (dDEGs) and upregulated DEGS (uDEGs) separately. For each TF, the enrichment was estimated based on the analysis of the 2 × 2 contingency table with the significance level calculated using Fisher’s exact test. To ensure a large-scale analysis, we took advantage of using a representative collection of DAP-seq data on TF binding in *A. thaliana* [16,32]. Recently, we have implemented this kind of enrichment analysis procedure as a CisCross-Main function within the CisCross web server [32].

**Figure 2:**
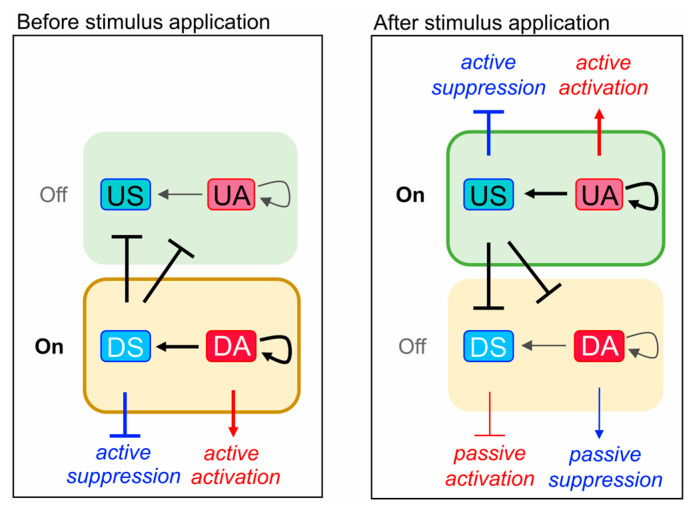
Two types of transcriptional subnetworks distinguished with FindTFnet. A repressed subnetwork (R-subnetwork, highlighted with a yellow box) operates before stimulus application, being switched off by a stimulus. An activated subnetwork (A-subnetwork, highlighted with a green box) is switched on by the stimulus. Self-activation links generalize UA–UA and DA–DA links. USes and DAs promote active and passive suppression of gene expression in response to the stimulus (blue arcs), while UAs and DSes promote active and passive activation of gene expression in response to the stimulus (red arcs). Bold arcs correspond to the regulatory links reinforced under the specified conditions. DA, Downregulated Activator; DS, Downregulated Suppressor; US, Upregulated Suppressor; UA, Upregulated Activator.

Second, we functionally characterize enriched TFs as transcriptional activators or suppressors. This step is crucial for providing mechanistic insight into the dynamics of TFRNs. Literature mining is not very effective for this purpose because some TFs act as transcriptional activators or suppressors depending on the conditions such as tissue type, environment, etc., [33]. To distinguish between transcriptional activators and suppressors in the context of certain experimental conditions, we offer a simple data-driven approach. It is reasonable to assume that a TF, whose binding peaks are enriched in uDEGs, acts as an activator if it is encoded by a uDEG (further, we call such TF an Upregulated Activator, UA), and acts a suppressor if it is encoded by a dDEG (Downregulated Suppressor, DS) (Figure 1B). Similarly, a TF, whose binding peaks are enriched in dDEGs, likely acts as a suppressor if it is encoded by a uDEG (Upregulated Suppressor, US), and it likely acts an activator if it is encoded by a dDEG (Downregulated Activator, DA) (other details see below in the Methods section). The four TF classes (US, UA, DA, DS) have the following interpretation. The US is a stimulus-induced suppressor. It inhibits expression of its target genes, which were active before the stimulus action (Figure 2). Conversely, the UA, which is induced by a stimulus, activates expression of its target genes. DAs and DSes were active in the absence of a stimulus (Figure 2). Upon stimulus application, DA expression decreases together with the expression of its target genes, thereby providing their passive suppression. Conversely, DSes suppressed the stimulus-responsive genes in the absence of the stimulus. Upon stimulus application, DS expression decreases, and its targets become deblocked, thereby being activated passively.

Third, of all detected DAs, DSes, USes and UAs, we select potential “TF regulator–TF target” pairs (hereafter referred to as TF pairs or links). Namely, we put a link from TF1 (regulator) to TF2 (target) if there is a TF1 binding peak in the 5′ regulatory region of TF2 coding gene. We consider such TF pairs as the elementary components of a TFRN induced by the stimulus. The link type (activation or inhibition) is assigned according to the predicted function of the TF regulator (activator or suppressor) (Figure 1A). Of 16 possible pairs between four TF regulation types, only eight (UA–UA, UA–US, DS–UA, DS–US, US–DA, US–DS, DA–DA, DA–DS) are meaningful. We visualize the resulting network as a graph. The employment of US/UA/DA/DS classification described above allows distinguishing two distinct subnetworks within the TFRN (Figure 2). The first one operates before the stimulus application, being switched off by the stimulus (hereafter referred to as a repressed network or R-subnetwork), and the second one is switched on by the stimulus (hereafter referred to as an activated network or A-subnetwork).

### 2.2. Auxin-Induced Reprogramming of Transcriptional Network in the A. thaliana Root

We used FindTFnet to reconstruct a TFRN from previously published microarray data on auxin-induced transcriptome in the roots of *A. thaliana* seedlings [34]. Inhibition of auxin transport prior to auxin treatment ensured a synchronization of lateral root initiation and other auxin-driven processes resulting in an elevated number of auxin DEGs (6704 dDEGs and 5201 uDEGs) [35] (Table S1), which were used as an input for FindTFnet. The DAP-seq data recruited by FindTFnet to map TF binding loci in *A. thaliana* genome contain two types of peak sets, which differ in the source genomic DNA libraries: (1) leaf gDNA possessing epigenetic DNA modifications (“col” data), and (2) leaf gDNA with methylcytosines eliminated due to PCR amplification (“colamp” data) [14]. Employment of TF binding loci in globally demethylated “colamp” genome for the analysis of auxin-sensitive DEGs yielded twice as many regulatory links compared to the native one (271 vs. 129). Wherein, the two sets of links poorly overlapped (29 TF pairs in common) (Table S2), pinpointing an extensive reprogramming of TF activity after DNA demethylation. Since even much weaker demethylation of DNA results in strong developmental defects in *A. thaliana* [36,37,38], and DNA methylation patterns in plants are quite similar in various vegetative tissues [39,40], we chose using “col” peak sets for further analyses of root transcriptomes.

**Figure 3:**
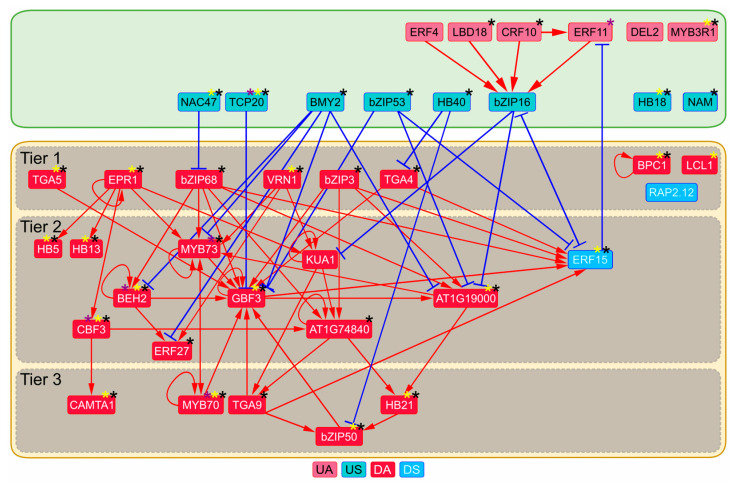
Auxin-regulated TFRN in *A. thaliana* roots predicted with FindTFnet. A repressed subnetwork (R-subnetwork) is highlighted with a yellow box, an activated subnetwork (A-subnetwork) is highlighted with a green box. Gray boxes denote three tiers of R-subnetwork. Black, yellow and purple asterisks mark the targets of activating ARFs, repressing ARFs and ARF3/ETTIN (repressing/activating), respectively, (see Table S2F for details). UA, upregulated activator; US, upregulated suppressor; DA, downregulated activator; DS, downregulated suppressor.

The binding of 60 TFs was enriched in 5′ regulatory regions of u/dDEGs (Table S2). Among those, 23 DAs, two DSes, eight USes and five UAs were detected. Moreover, 16% of TFs including one DA (LCL1), one DS (RAP2.12), two UAs (MYB3R1 and DEL2), and two USes (HB18 and NAM) were not a part of any “TF regulator–TF target” pair; one DA (BPC1) was only self-activated (Table S2). The rest of the regulators made up a connected transcriptional network (Figure 3). The robust TFRN nodes persisting when the fold change threshold of DEG calling increases are depicted in the Supplementary Figure. The reconstructed TFRN is divided into two subnetworks (Figure 3). The preexisting subnetwork, repressed by auxin upon its application (R-subnetwork), is quite extensive and is represented by an extensive DA–DA part and two DSes. One of the DSes, ERF15, is redundantly activated by numerous DAs. The DA–DA part falls into three tiers. Tier 1 contains the nodes with only outgoing arcs within the subnetwork (excluding EPR1 self-regulation), tier 2 includes the nodes triggered by those from tier 1, and tier 3 combines the rest of the nodes. The subnetwork comprises numerous feedback and feedforward loops; self-activation was predicted for seven DAs (BEH2, EPR1, GBF3, KUA1, MYB70, MYB73 and AT1G74840). ERF15 represses R-subnetwork transition to the “off” state. Noteworthy, the nodes with the highest number of outgoing edges (bZIP68, EPR1, bZIP3, and VRN1, which activate six, five, four, and three TF-coding genes, respectively) are limited to tier 1 DAs, and likely act as triggers in the R-subnetwork. A-subnetwork is less extensive (Figure 3). However, a few USes directly downregulate expression of about 40% of TF-coding genes in the R-subnetwork (including *bZIP68*, one of the predicted transcriptional triggers, and *ERF15*, the repressor of the R-subnetwork inactivation). The genes encoding seven USes (all but bZIP16) and three UAs (LBD18, CRF10, MYB3R1) are potential targets of activating ARFs according to ChIP-seq data (Figure 3, Table S2F). Similarly, one DS (ERF15) and 15 DAs (all but bZIP3, bZIP68, TGA4, TGA9, MYB73, KUA1, ERF27, AT1G74840) are potential targets of repressing ARFs. Thus, auxin response likely involves numerous feedforward loops. Moreover, ERF11 (UA), bZIP16 (US) and ERF15 (DS) are the candidate participants of feedback/feedforward loops between two subnetworks. The reconstructed TFRN allows assuming that extensive reprogramming of the preexisting transcriptional network plays an essential role in response to auxin treatment.

### 2.3. The Functions of Some TFs within the TFRN May Depend on the Co-Occurrence of Their Binding Loci in Target Promoters

It is worth noting that the regulatory mode reconstructed for each TF within the TFRN is an integrated characteristic, determined by a complex of conditions. To gain a deeper insight into the nature of this integrated characteristic, we assessed how well the predicted TF activities correlated with the experimentally established ones based on the published data. Among others, we used a recent large-scale study of the transcriptional effector domain (TED) activities for over 400 *A. thaliana* TFs [41]. The role of the predicted activators was well confirmed. Of 23 DAs and six UAs, the activator function was supported for 14 (61%) and six (100%) TFs, respectively; for two DAs no experimental data were found (Table 1 and Table S3A). In contrast, five of eight USes (63%) have been previously reported as activators only. Similarly, we did not find any data confirming the repressor function of the predicted DSes. Noteworthy, not a single TF with a known repression motif or with an established repressor activity of TED according to [41] was found among the predicted suppressors. Therefore, we assumed that the predicted suppressors inhibit transcription indirectly, by competing with transcriptional activators for cognate DNA-binding sites or by recruiting co-repressors (including other TFs) as suggested in [42]. Indeed, both activator and repressor functions have been previously reported for three predicted DAs (bZIP68, MYB73, TGA4), two UAs (DEL2, MYB3R1), and one US (TCP20), which have no repression domains detected (Table S3A), as well as for many other TFs known as typical transcriptional activators [43,44,45,46]. The same mechanisms can explain the predicted activator role of five DAs (BPC1, EPR1, HB5, HB21, VRN1), which lack known repression motifs but were identified in previous publications only as transcriptional repressors (Table S3A).

**Figure 4:**
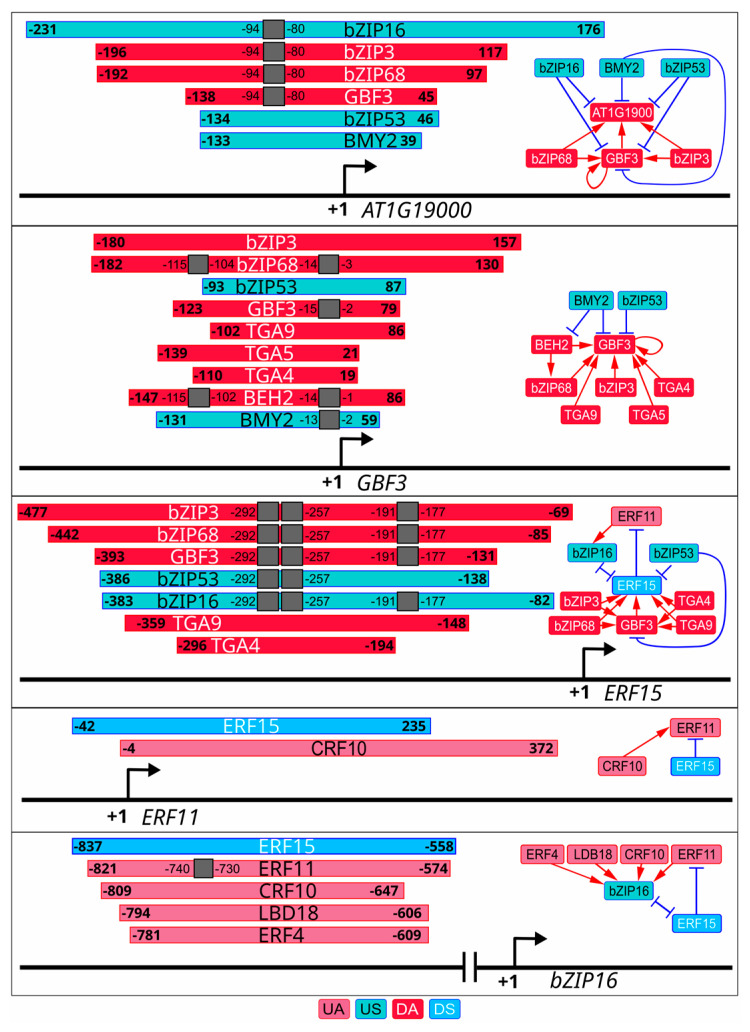
Co-occurrence of TF regulators’ binding loci in the promoters of their TF-coding targets. DAP-seq peaks are depicted as colored boxes with sharp corners, the positions of TF binding sites are marked as gray boxes. The numbers denote the peak/motif coordinates relative to the transcription start site. The box color codes the type of the regulator (UA, US, DA, DS), which binds in the corresponding locus. The regulatory relationships between TF-encoding gene and its regulators, reflected in TFRN, are represented on the right. UA, upregulated activator; US, upregulated suppressor; DA, downregulated activator; DS, downregulated suppressor.

To investigate the hypothesis about the competitive/cooperative mechanisms of gene repression by some of the predicted regulators involved in auxin response, we investigated the co-localization of TF binding loci within the promoters of TF-coding genes in TFRN. We found that DAP-seq peaks for the regulators often stacked in the promoters (Figure 4). Noteworthy, binding within the same region was characteristic both for the members of the same TF family (e.g., bZIP TFs in the promoters of *AT1G19000* and *ERF15*; ERF/AP2 TFs in the *ERF11* promoter) and for unrelated TFs (e.g., ERF/AP2 and LBD TFs in the promoter of *bZIP16*; bZIP and BES1/BZR1 TFs in the *GBF3* promoter) (Figure 4, Table S3B). Therefore, it is reasonable to assume that, in response to auxin, USes replace DAs and occupy their position in the promoters of dDEGs (e.g., *AT1G19000*, *GBF3*, *ERF15*). At that, DA replacement with a US, which is in fact a weaker activator, would manifest as a transcriptional repression. This can be the most likely scenario for DA/US regulator pairs with both TFs belonging to the same family and sharing a common binding sequence. Similarly, UAs can possibly replace DSes in the promoters of uDEGs (e.g., *bZIP16*, *ERF11*). At that, a weaker activator being replaced with a stronger one would manifest itself as a transcriptional repressor. This scenario explains the lack of the repression domains in the predicted suppressors quite well. Another possible option is a cooperative repression of gene expression by co-bound TF-activators, as it has been previously shown for *A. thaliana* ARR18 and bZIP63: both TFs bind to *PDH1* promoter, and interaction between TFs blocks bZIP63 activator capacity [47]. The likely candidates are USes or DSes from different families, which co-bind in the promoters (e.g., USes from bZIP and BES1/BZR1 families in *GBF3* promoter) (Figure 4). It is also worth considering that possible repressor domain-containing TFs, which may cooperate with the predicted regulators, may be auxin-insensitive (and therefore excluded from our analysis) or missing in the DAP-seq library recruited by FindTFnet.

An intriguing finding was that four predicted DAs (MYB70, TGA5, AT1G19000 and KUA1) and two UAs (ERF4 and ERF11) possessed an intrinsic repression motif [48,49,50,51] and/or demonstrated a repressor activity of TED according to [41] (Table S3A). This raises the question of possible mechanisms, which could promote gene activation in these cases. The capability to enhance transcription was previously reported for ERF4, ERF11, MYB70 and TGA5 (Table S3A). One option is an expression of a short TF isoform lacking the repression motif as in the case of ERF4 [50]. Another possibility is a transcriptional regulation in cooperation with other TFs as it is supposed to happen in the case of MYB70 [48]. Accordingly, we observed co-binding of the above-mentioned regulators with TFs from distinct families in the promoters (e.g., ERF4/11 and LBD18 in promoter of *bZIP16*, Figure 4). Thus, the TFRN provides the possibility to generate hypotheses on molecular mechanisms of TF functions for further studies.

### 2.4. Auxin-Regulated TFRN Is Associated with Biological Processes Affected by Auxin Treatment

To get insight into the mechanisms, which link auxin treatment to physiological traits in *A. thaliana* roots, we investigated involvement of the auxin TFRN in regulation of biological processes (BPs) enriched in DEGs. We found 34 and 49 BPs enriched in dDEGs and uDEGs, respectively. Particularly, auxin downregulated circadian rhythm; autophagy; photosystem II assembly; vesicle mediated and intracellular protein transport; cell wall organization or biogenesis; epidermis development; abscisic acid (ABA) signaling; lignin, chlorophyll and glucosinolate biosyntheses and responses to water deprivation, light intensity, wounding, oxidative and salt stresses, cold and bacteria (Table S4A). Auxin upregulated ribosome biogenesis; cell division; protein folding; protein import into nucleus and mitochondria; rRNA and mRNA processing; cytoplasmic translation; mRNA transport; RNA modification; mitochondrion organization (Table S4B). Unexpectedly, gene ontology (GO) terms associated with embryo and seed development (embryo development ending in seed dormancy, embryo sac development and seed development) were enriched in uDEGs identified in roots. We found that enrichment of these GO terms is provided by the genes, which, being critical regulators of embryo and/or seed development, continue to play an essential role in regulation of other BPs throughout entire life cycle of the plant, for example, *PIN FORMED 1* (*PIN1*), which encodes auxin efflux carrier [52]. In total, 2706 dDEGs and 1693 uDEGs were associated with auxin down- and upregulated processes, respectively. Since USes and DAs promote active and passive suppression of genes in response to auxin, while UAs and DSes promote their active and passive activation (Figure 2), we further focused on the involvement of USes/DAs and UAs/DSes in regulation of dDEGs and uDEGs associated with auxin-affected BPs, respectively.

#### 2.4.1. Auxin-Dependent Repression of Biological Processes

As many as 1983 dDEGs (73%) were potential targets of DAs and/or USes. At the level of individual BPs, each downregulated process was tightly linked to USes/DAs as well. Thus, the fraction of potential DA targets among BP-associated dDEGs varied from 55% in glucosinolate biosynthesis to 84% in photosynthesis (Table S5A). Moreover, DAs were extensively involved in their regulation: of 23 DAs, 10 appeared as potential auxin-dependent regulators in all 34 BPs enriched in dDEGs, while 11 appeared in all except for no more than three BPs (Table S5B). At the same time, the fraction of potential US targets among BP-associated dDEGs was less than observed for DA targets: it varied from 15% in autophagy to 41% in circadian rhythm with an average of 28% (Table S6A). Taking into account that a high fraction of DAs (42%) are potential US targets (Figure 3), we assume that active suppression by USes makes a critical contribution to attenuation of R-subnetwork, which maintains BPs prior to auxin treatment. This attenuation, in turn, predominantly mediated downregulation of the rest of the genes involved in control of the BPs. Despite the (predicted) minor role of active repression in auxin-dependent attenuation of non-TF-coding genes, in one way or another USes affected most downregulated BPs: they appeared as potential regulators in 23 to 34 BPs downregulated by auxin (Table S6B). Some genes were extensively regulated by DAs and USes. Thus, depending on BP, the number of potential DA-regulators of one gene could reach up to 13 as it was observed for responses to abscisic acid, blue light and cold (Table S7A). In the case of USes, this value was up to five (Table S7B). Importantly, such extensively regulated genes often play a key role in the BP (see the Discussion section for the details).

**Figure 5:**
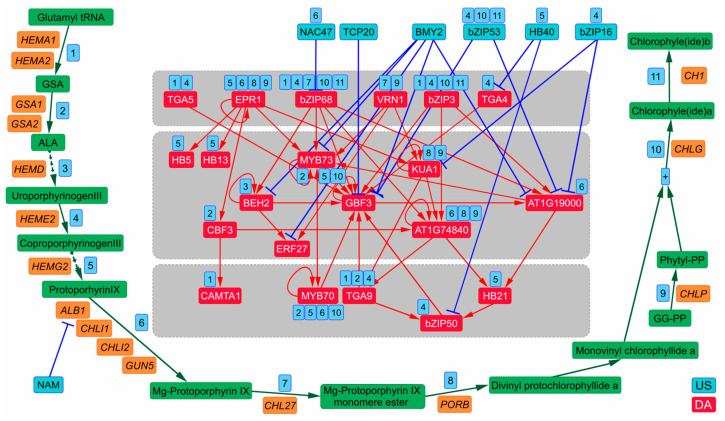
Auxin-regulated TFRN attenuates chlorophyll biosynthesis. Green filled boxes stand for the substrates/products of the reactions involved in the chlorophyll biosynthesis pathway. Green arrows denote chemical transformations. Orange filled boxes signify dDEGs, which encode chlorophyll biosynthesis enzymes. TFRN is represented by its interconnected US–DA part supplemented with the free-standing US NAM, which is the regulator of one dDEG encoding a chlorophyll biosynthesis enzyme. The numbers in blue boxes denote ordinal numbers of the steps in the chlorophyll biosynthesis pathway (only the ones affected by USes/DAs are represented). Next to a TF, these numbers indicate that the TF regulates chlorophyll biosynthesis genes at the corresponding step. A “plus” sign in a blue box denotes entering of an additional chain of reactions to the main one. US, upregulated suppressor; DA, downregulated activator. Abbreviations for the chlorophyll biosynthesis genes (or genes/enzymes if gene and enzyme names differ): *ALBINA 1*/Magnesium chelatase (*ALB1*/MgMT), *CHLORINA 1*/Chlorophyllide a oxygenase (*CH1*/CAO), *CHLOROPHYLL 27/*Mg protoporphyrin IX monomethylester cyclase *(CHL27/*MgMT), *CHLOROPHYLL G/*Chlorophyll synthase (*CHLG/*CHLG), *CHLOROPHYLL I1/*Magnesium chelatase (*CHLI1*/MgMT), *CHLOROPHYLL I2/*Magnesium chelatase (*CHLI2*/MgMT), *GENOMES UNCOUPLED 5/*Magnesium chelatase (*GUN5*/MgMT), *HEME A1/*Glutamyl tRNA reductase (*HEMA1/*GluTR), *HEME A2/*Glutamyl tRNA reductase (*HEMA2/*GluTR), *HEME D/*Uroporphyrinogen III synthase (*HEMD/*UROS), *HEME E2/*Uroporphyrinogen III decarboxylase (*HEME2/*UROD), *HEME G2/*Protoporphyrinogen oxidase (*HEMG2/*PPOX), *GLUTAMATE 1 SEMIALDEHYDE 2,1 AMINOMUTASE 1/*Glutamate 1-semialdehyde 2,1 aminomutase (*GSA1*/GSA-AT), *GLUTAMATE 1 SEMIALDEHYDE 2,1 AMINOMUTASE 2/*Glutamate 1 semialdehyde 2,1 aminomutase (*GSA2*/GSA-AT), *PHYTYL CHLOROPHYLL/*Geranylgeranyl reductase (*CHLP*/GGR), *PROTOCHLOROPHYLLIDE OXIDOREDUCTASE B* (*PORB*). Abbreviations for the substrates: 5-aminolevulinic acid (ALA), Glutamate 1-semialdehyde (GSA), Phytyl diphosphate (Phytyl-PP), geranylgeranyl-diphosphate (GG-PP).

**Figure 6:**
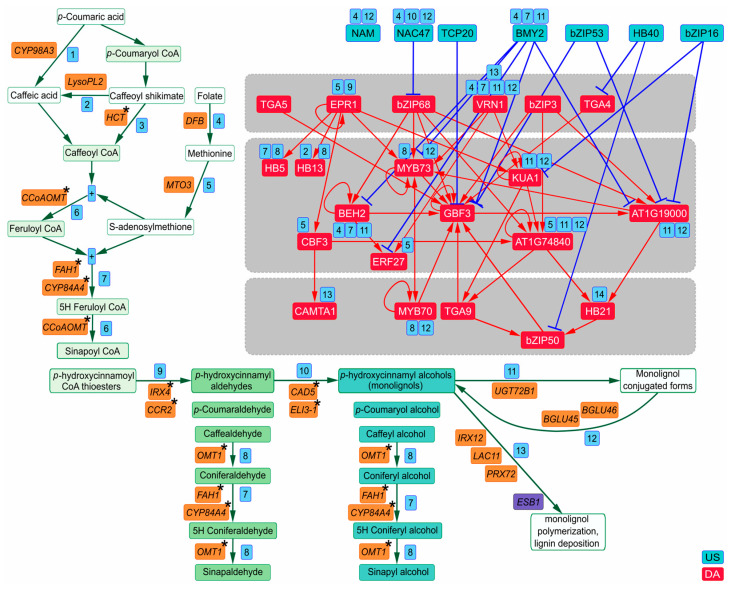
Auxin-regulated TFRN attenuates lignin biosynthesis. Green boxes stand for the substrates/products of the reactions involved in lignin biosynthesis. Green arrows denote chemical transformations. Orange and violet filled boxes present the genes encoding lignin biosynthesis enzymes and their regulators, respectively. First, *p*-Coumaric acid is transformed into several *p*-hydroxycinnamoyl CoA thioesters (highlighted in pastel mint), which are interconverted by CCoAOMT and F5H. Second, *p*-hydroxycinnamoyl CoA thioesters are transformed to *p*-hydroxycinnamyl aldehydes (highlighted in light green) by CCR, which are further converted to *p-*hydroxycinnamyl alcohols (monolignols) (highlighted in aquamarine) by CAD. *p*-hydroxycinnamyl aldehydes and monolignols interconversion is provided by COMT and F5H. Finally, monolignols polymerize into lignin. UGT72B1 catalyzes monolignol conjugate formation, and BGLU45 and BGLU46 promote their deconjugation. Laccases, peroxidases and ESB1 enable monolignols’ polymerization to lignin and its deposition. TFRN is represented by its interconnected US–DA part supplemented with the free-standing US NAM, which is a regulator of two dDEGs encoding lignin biosynthesis enzymes. The numbers in blue boxes mark the steps in the lignin biosynthesis pathway (only the ones affected by USes/DAs are represented). Next to a TF, these numbers indicate that the TF regulates lignin biosynthesis genes at the corresponding step. A “plus” sign in a blue box denotes entering of an additional chain of reactions to the main one. Asterisks point out the genes activated by MYB63 [57]. US, upregulated suppressor; DA, downregulated activator. Abbreviations for the lignin biosynthesis genes (or genes/enzymes if gene and enzyme names differ): *HYDROXYCINNAMOYL:COA SHIKIMATE HYDROXYCINNAMOYL TRANSFERASE* (*HCT*); *CYTOCHROME P450*, *FAMILY 98*, *SUBFAMILY A*, *POLYPEPTIDE 3*/Coumaric acid 3-hydrolase (*CYP98A3*/C3H); *LYSOPHOSPHOLIPASE 2*/Caffeoyl shikimate esterase (*LysoPL2*/CSE); *CAFFEOYL COA O-METHYLTRANSFERASE* (*CCoAOMT*); DHFS-FPGS HOMOLOG B/Folylpolyglutamate synthetase 1 (*DFB*/FPGS1); *METHIONINE OVER-ACCUMULATOR 3*/S-adenosylmethionine synthetase 3 (*MTO3*/SAMS3); *FERULIC ACID 5-HYDROXYLASE 1*/FERULATE 5-HYDROXYLASE 1 (*FAH1*/F5H1); *CYTOCHROME P450*, *FAMILY 84*, *SUBFAMILY A*, *POLYPEPTIDE 4*/FERULATE 5-HYDROXYLASE 2 (*CYP84A4*/F5H2); *O-METHYLTRANSFERASE 1*/Caffeic acid O-methyltransferase 1 (*OMT1*/COMT1); *IRREGULAR XYLEM 4*/Cinnamoyl CoA reductase 1 (*IRX4*/CCR1); *CINNAMOYL COA REDUCTASE 2* (*CCR2*); *CINNAMYL ALCOHOL DEHYDROGENASE 5* (*CAD5*); *ELICITOR-ACTIVATED GENE 3-1*/Cinnamyl alcohol dehydrogenase 7 (*ELI3-1*/CAD7); *BETA-GLUCOSIDASE* (*BGLU*); *UDP-GLYCOSYLTRANSFERASE 72B1* (*UGT72B1*); *IRREGULAR XYLEM 12*/Laccase 4 (*IRX12*/LAC4); *LACCASE* (*LAC*); *PEROXIDASE 72* (*PRX72*/PER72); *ENHANCED SUBERIN 1* (*ESB1*); *MYB DOMAIN PROTEIN 63* (*MYB63*).

**Figure 7:**
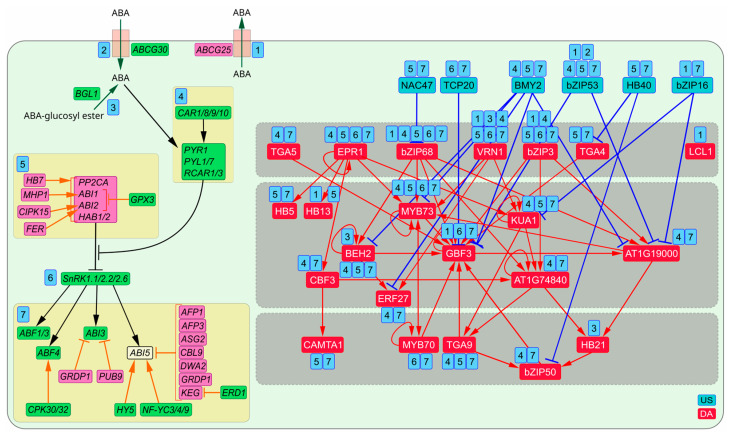
Auxin-regulated TFRN attenuates ABA transport, conjugation and signaling. Green and pink filled boxes signify dDEGs, which encode activators and repressors of ABA signaling, respectively. Black and orange arrows/bar-headed lines on the left scheme depict ABA signaling pathway and its transcriptional regulation, respectively. TFRN is represented by its interconnected US–DA part supplemented with the free-standing DA LCL1, which is a regulator of a dDEG encoding ABA transporter. The numbers in blue boxes denote ordinal numbers of the steps in the ABA signaling. Next to a TF within the TFRN, these numbers indicate that the TF regulates the corresponding step. US, upregulated suppressor; DA, downregulated activator. Abbreviations for the ABA transport, conjugation and signaling genes: *ATP-BINDING CASETTE G25/30* (*ABCG25/30*), *β-GLUCOSIDASE HOMOLOG 1* (*BGL1*), *PYRABACTIN RESISTANCE1/PYR1 LIKE/REGULATORY COMPONENTS OF ABA RECEPTORS* (*PYR/PYL/RCARs*), *C2-DOMAIN ABA-RELATED* (*CAR*), *PROTEIN PHOSPHATASES TYPE 2C* (*PP2CA*), *ABA INSENSITIVE1/2/3/5* (*ABI1/2/3/5*), *HYPERSENSITIVE TO ABA1/2* (*HAB1/2*), *HOMEOBOX 7* (*HB7*), *CALCINEURIN B-LIKE PROTEIN-INTERACTING PROTEIN KINASE15* (*CIPK15*), *FERONIA* (*FER*), *THE YEAST MPO1 HOMOLOG IN PLANTS* (*MHP1*), *GLUTATHIONE PEROXIDASE 3* (*GPX3*), *SNF1-RELATED PROTEIN KINASE 1* (*SnRK1*), *ABSCISIC ACID RESPONSIVE ELEMENT-BINDING FACTOR1/3/4* (*ABF1/3/4*), *CALCIUM-DEPENDENT PROTEIN KINASE 30/32* (*CPK30/32*), *CALCINEURIN B-LIKE PROTEIN 9* (*CBL9*), *ALTERED SEED GERMINATION 2* (*ASG2*), *ABI FIVE BINDING PROTEIN 1/3* (*AFP1/3*), *DWD HYPERSENSITIVE TO ABA2* (*DWA2*), *KEEP ON GOING* (*KEG*), *ENHANCED DISEASE RESISTANCE 1* (*EDR1*), *PLANT U-BOX/ARM-REPEAT* (*ATPUB-ARM*) *E3 LIGASE 9* (*PUB9*), *GLYCINE-RICH DOMAIN PROTEIN 1* (*GRDP1*), *ELONGATED HYPOCOTYL 5* (*HY5*), *NUCLEAR FACTOR Y3/4/9* (*NF-YC3/4/9*).

For a deeper insight into the mechanisms, employed by auxin to downregulate specific BPs, we overlaid TFRN on the pathways for chlorophyll biosynthesis (which takes place in roots under light exposure [53,54]), lignin biosynthesis and ABA transport, conjugation and signaling (Figure 5, Figure 6 and Figure 7), attenuated by auxin according to our functional annotation. While auxin-dependent downregulation of chlorophyll and lignin biosynthesis was previously shown [53,54,55,56,57], only positive regulation of ABA signaling by auxin was described before [58,59]. 21 dDEGs associated with chlorophyll biosynthesis encode the vast majority of its enzymes (Table S9A, Figure 5). In line with the trend outlined above, the USes/DAs provide a very tight control of chlorophyll biosynthesis: 16 (76%) dDEGs encoding chlorophyll biosynthesis enzymes are potential targets of USes/DAs (Figure 5, Table S8A). Of them, *HEME2* is a hub targeted by eight bZIP family TFs (Table S8B), *HEMG2* is a target of seven TFs (four HD-ZIP and three MYB family TFs), five TFs (three bZIP and two MYB family TFs) bind in *CHLG* promoter, three dDEGs, *ALB1*, *GSA1* and *CHLP*, are regulated by four TFs each. Moreover, 78% of DAs and 63% of USes are involved in regulation of chlorophyll biosynthesis genes (Figure 5, Table S8B). Ten USes/DAs regulate more than one step in chlorophyll biosynthesis pathway, wherein, DAs activate up to five different steps (bZIP68), and USes suppress up to three steps (bZIP53). bZIP3, bZIP68 not only trigger the TFRN but directly control steps at both the beginning and the end of chlorophyll biosynthesis.

Lignin is an aromatic heteropolymer, which makes cell walls rigid and hydrophobic [60,61,62]. Lignin is produced during secondary cell wall thickening in development and in some stress responses as a result of an oxidative polymerization of monolignols mediated by laccases and/or peroxidases (Figure 6). Monolignols (*p*-coumaryl, coniferyl, and sinapyl alcohols) are synthesized through a phenylpropanoid biosynthetic pathway, which consists of three main phases (Table S9A, Figure 6) [62,63,64]. dDEGs contain 20 genes, which encode the majority of enzymes for lignin biosynthesis, as well as two essential regulators of lignin deposition, ESB1 and MYB63 (Table S9A, Figure 6). Of those, 16 dDEGs (73%) were predicted as direct targets of USes/DAs (Table S9A). The process of monolignol conjugation and deconjugation is regulated most redundantly: genes encoding BGLU45/46, the enzymes producing free monolignols from their conjugated forms, monolignol glucosides [65], and UGT72B1, the enzyme producing conjugated monolignols [66], are the potential targets of eight and six TFs, respectively, (Figure 6, Table S9B). In methylation of *p*-hydroxycinnamoyl CoA thioesters, *DFB*, which links one-carbon metabolism to lignin biosynthesis [67], and *MTO3* [68] have correspondingly five and four TFs directly regulating their activity. Each interconversion reaction of *p*-hydroxycinnamyl aldehydes and monolignols is regulated by four TFs. Lignin polymerization and *p*-hydroxycinnamoyl CoA thioesters conversion into *p*-hydroxycinnamyl aldehydes are under three and two TFs, respectively. Thus, as in the case of chlorophyll biosynthesis the TFRN provides the very tight control of lignin biosynthesis. Altogether, 80% DAs and 50% USes directly trigger genes encoding enzymes for this process (Figure 6, Table S9B). Among DAs, VRN1 activates five different steps in lignin biosynthesis. AT1G74840 and BEH2 each upregulate enzyme-coding genes in three steps. DAs AT1G19000, KUA1, HB5, HB13, and EPR1 keep active two steps of lignin biosynthesis each. Auxin switches on USes NAC47 and BMY2, which directly suppress enzyme-coding genes that enable three different steps of lignin biosynthesis. The US NAM directly downregulates two lignin biosynthesis steps. Thus, the tight TFRN control of lignin biosynthesis is also highly coordinated; at least twelve TFs within the TFRN regulate more than one lignin biosynthesis step each.

dDEGs contain 43 genes encoding ABA signaling components and their regulators (Table S10, Figure 7). Among them, ABCG25 and ABCG30 regulate ABA levels in cells by ABA transport [69,70]. BG1 hydrolyzes glucose-conjugated ABA increasing ABA level [71]. ABA binds to and activates PYR/PYL/RCAR receptors PYR1, PYL1, PYL7, RCAR1, and RCAR3 [72]. CAR proteins enhance PYR/PYL/RCAR activity [73]. PYR/PYL/RCARs inhibit PP2Cs, PP2CA, ABI1, ABI2, HAB1 and HAB2. HB7 activates transcription of their genes [74]. CIPK15 and FER activate the ABI2-coding gene [75,76], whereas MHP1 enhances *ABI1* transcription [77]. GPX3 inhibits both *ABI1* and *ABI2* [78]. Thus, when PYR/PYL/RCARs prevent PP2Cs from dephosphorylating SnRKs, they transphosphorylate their targets [72,73]. Among them, there are ABF1, ABF3, ABF4, ABI3 and ABI5. CPK30 and CPK32 activate ABF4 by phosphorylation [79]. CBL9, AFP1, AFP3, DWA2 and KEG suppress *ABI5* [80,81,82,83,84,85]. Most of them promote degradation of ABI5 protein. EDR1 decreases KEG activity, which, in its turn, increases the ABI5 level [86]. PUB9 promotes ABI3 degradation [87]. GRDP1 inhibits both ABI5 and ABI3 [88]. TFs HY5, NF-YC3, NF-YC4 and NF-YC9 activate *ABI5* [89,90,91]. 37 dDEGs (79%) encoding ABA signaling components and their regulators are potential targets of the TFRN (Figure 7, Table S10A). Among them, there are *PYL7*, *NF-YC9* and *ABCG25* that are regulated by 12, 11 and nine TFs, respectively (Table S10B). ABI5 inhibitors *AFP3* and *CBL9*, ABI2 activator *CIPK15*, and ABI3 inhibitor *PUB9* are targeted by seven TFs. Four TFs monitor ABF3 *ABI1*, *AFP1* and *SNRK2.2* activity. Thus, the TFRN provides very tight control of ABA signaling. All interconnected DAs and USes, as well as free standing LCL1 compose the TFRN targeting genes encoding components of ABA signaling (Figure 7). The main triggers of the TFRN (VRN1, bZIP68, bZIP3 and EPR1) activate 9, 12, 10 and 7 components of ABA signaling, respectively. By this way VRN1 controls six stages in ABA signaling, bZIP68 and bZIP3 each maintain activity at five stages and EPR1 at four stages. Members of the second tier in the cascade MYB73 and BEH2 promote four stages. Other TFs ensure upregulation of three or two stages. LCL1 and HB21 specifically regulate ABA efflux and ABA deconjugation, respectively. Among USes, bZIP53 directly suppresses seven components at five different stages of ABA signaling, BMY2—six components at three stages. Other USes block each two different stages. VRN1, bZIP3 and bZIP68 not only trigger the TFRN but are the master coordinators of ABA signals guiding both early and last steps in this process. Thus, the tight TF control of ABA signaling is also highly coordinated, at least most TFs within the TFRN regulate more than one step in it. The majority of TFs modulate the ABA signal by regulating both activators and suppressors of ABA signaling (Figure 7).

#### 2.4.2. Auxin-Regulated TFRN Controls Activation of Ribosome Biogenesis

Only 485 uDEGs associated with auxin-affected BPs (29%) were potential targets of DSes and/or UAs. For eight BPs, no potential DS target genes have been found. In other BPs, 3% to 22% genes were potential direct DS targets (Table S11). UAs directly enhance activity from 6% of genes in RNA methylation to 58% in microtubule-based movement (an average of 28%) (Table S12A). In most of the BPs a gene is upregulated by only one TF (Table S12). About one third of BPs are upregulated by all UAs (Table S13).

**Figure 8:**
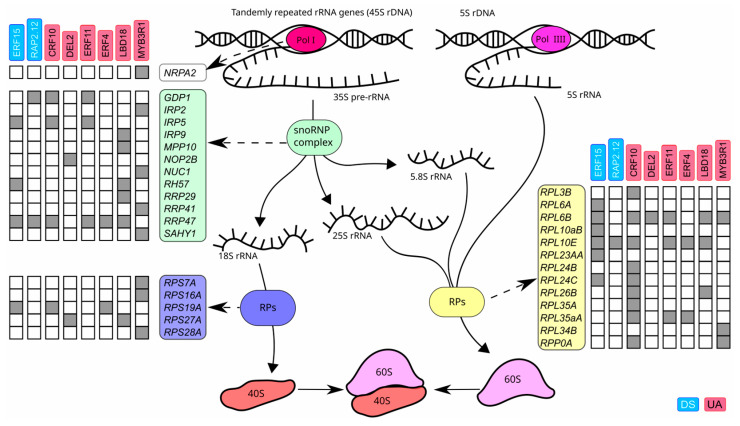
Auxin-guided TFRN upregulates ribosome biogenesis and activity of several genes encoding components for this process. Schematic representation of functions in the ribosome biogenesis for uDEGs enriched in this GO term and the TFs enhancing their activity. RPs—ribosomal proteins. Gray colored squares mark the targets of DSes and UAs from the TFRN induced by auxin treatment. Proteins involved in four steps of ribosome biogenesis are enclosed in frames with a white (1), light mint (2), blue (3) and yellow (4) background. TFs regulating transcription of uDEGs encoding these proteins are given in the tables located on the left of the proteins of the rRNA processing complex and ribosomal proteins forming the small ribosomal subunit and on the right of the ribosomal proteins forming the large ribosomal subunit. UA, upregulated activator; DS, downregulated suppressor. Abbreviations for the ribosome biosynthesis genes and enzymes: Polymerase I/III (Pol I/III), *NUCLEAR RNA POLYMERASE A2* (*NRPA2*), *G-PATCH DOMAIN PROTEIN 1* (*GDP1*), *INVOLVED IN RRNA PROCESSING* (*IRP*), *RIBOSOMAL RNA processing PROTEIN* (*RRP*), RIBOSOMAL PROTEIN SMALL subunits (RPS), RIBOSOMAL PROTEIN LARGE subunits (RPL), 60S acidic ribosomal protein P0 (RPP0A), *RNA HELICASE 57* (*RH57*), *NUCLEOLAR RNA-BINDING PROTEIN 2B* (*NOP2B*), *M PHASE PHOSPHOPROTEIN 10* (*MPP10*), *NUCLEOLIN LIKE 1* (*NUC1*), *SALT HYPERSENSITIVE 1* (*SAHY1*).

In functional annotation we found the following BPs related to ribosome biogenesis: rRNA processing; maturation of LSU-rRNA from tricistronic rRNA transcript (SSU-rRNA, 5.8S rRNA, LSU-rRNA); maturation of SSU-rRNA from tricistronic rRNA transcript (SSU-rRNA, 5.8S rRNA, LSU-rRNA); maturation of LSU-rRNA; maturation of 5.8S rRNA; maturation of SSU-rRNA; ribosomal large subunit assembly; ribosomal large subunit biogenesis; ribosomal small subunit assembly and ribosome biogenesis itself (Table S4B). The ribosome is a ribonucleoprotein complex, whose biogenesis starts from transcription of tandemly repeated rRNA genes [92]. In *A. thaliana*, 5S RNAs and 35S pre-rRNAs are transcribed by Polymerase III in nucleoplasm and Polymerase I in nucleolus, respectively. Polymerase I consists of 14 protein subunits, of which 12 subunits are homologous or common to those of other RNA polymerases and two subunits, NRPA1 and NRPA2, are Polymerase I specific [93]. 35S precursor transcript is then processed into the 18S, 5.8S and 25S rRNAs by ribonucleoprotein complex consisting of small nucleolar RNAs (snoRNAs) and proteins [92]. 18S rRNAs with ribosomal proteins form the small ribosomal subunit (40S). 5.8S and 25S/28S rRNAs give rise to the large ribosomal subunit (60S). We subdivided ribosome biogenesis into four main steps, which are clearly separated (Table S14, Figure 8): (1) transcription of rRNA genes, (2) processing of the primary transcript, (3) small ribosomal subunit assembly and (4) large ribosomal subunit assembly. We checked the published papers and each gene affiliation to the step is confirmed by experiment with the corresponding reference (Table S14A). Some genes are involved in processes at several steps, in this case the step, from which they started to be active, is mentioned.

uDEGs contain 161 genes encoding proteins for ribosome biogenesis. Most of them (146 genes) participate in biogenesis of cytoplasmic ribosomes, and only six and nine genes in mitochondrial and plastid ribosomes, respectively, (Table S14). Of them, 35 (22%) are the targets of six UAs and/or two DSes (Table S14B, Figure 8). Nevertheless, only MYB3R1 (UA) regulates step 1 of ribosome biogenesis targeting *NRPA2*, which encodes the specific Polymerase II subunit (Figure 8). MYB3R1 also alone guides expression of four, three and one genes participating, respectively, in steps 2, 3 and 4. In addition, MYB3R1 monitors ribosome biogenesis in mitochondria and plastids targeting *MITOCHONDRIAL RNA PROCESSING 1* (*MRP1/POP1*) and *REGULATOR OF FATTY-ACID COMPOSITION 3* (*RFC3*), respectively, (Table S14). LBD18 (UA) controls alone upregulation after auxin treatment four genes at the step 2 and one gene in plastid ribosome biogenesis, whereas CRF10 (UA) enhances alone five genes at the step 4 and one gene in mitochondria ribosome biogenesis. Some of these genes are inhibited in norm by ERF15 (DS). Steps 2 and 4 have the most targeting proteins having six links from DSes and/or UAs, namely for the step 2 it is RRP47, and for the step 4 RIBOSOMAL PROTEIN, LARGE subunits 10E (RPL10E) and 6B (RPL6B).

## 3. Discussion

### 3.1. Auxin TFRN Comprises Both Primary and Secondary ARF Targets

Reconstruction of TFRN with FindTFnet led to successful findings of TFs playing important roles in auxin regulation of biological processes. We focus on identification of TFs acting upstream auxin-induced DEGs, and these TFs can be not the primary ARF targets. In *A. thaliana* and maize, it was shown by comparison of the list of genes bound by a TF in ChIP-seq experiments with the list of DEGs from the TF perturbations (in lines with loss and/or gain-of function this TF), that only 20–30% of DEGs are primary targets of the TF [26,27]. Such a weak overlap allows suggesting that most DEGs are secondary or tertiary targets of the regulator(s) of transcriptional response. They may also be the transient targets not detectable bound by the master TF and not determined by conventional ChIP-seq and DAP-seq methods [94]. For NIN-LIKE PROTEIN 7 (NLP7), the master TF of nitrate response, the transient targets capture 50% of NLP7 regulated genes. Also our approach is not capable of univocally characterizing enriched TFs encoded by auxin-insensitive genes (not DEGs), and the ones enriched both in uDEGs and in dDEGs, as activators or suppressors, therefore, we filter them out from further analysis. However, it is worth remembering that these TFs could also participate in auxin response being induced post-transcriptionally, cell-type-specifically, at other developmental stages or as conditional activators or repressors [12,95]. Due to this, the TFRN presented in this issue is incomplete. The more complete auxin TFRN can be a superposition of several TFRNs, for example, operating in distinct tissues.

### 3.2. For Several TFRN TFs the Key Role in Auxin Response Have Been Previously Shown

The reconstructed TFRN generalizes a set of preceding results. Previously, the role in mediating auxin response was shown only for 13 TFs (22%) from the reconstructed TFRN. HB5 negatively regulates *AUXIN/INDOLE-3-ACETIC ACID 12/BODENLOS* (*IAA12/BDL*) expression and by this way modulates auxin response [96]. KUA1/MYBH enhances hypocotyl elongation by increasing auxin biosynthesis [97]. KUA1/MYBH suppresses auxin conjugation by direct binding to *DWARF IN LIGHT 1/GRETCHEN HAGEN3.6* (*DFL1/GH3.6*) and *DFL2/GH3.10* promoters [98], whereas MYB70 directly upregulates the expression of auxin conjugation-related GH3 genes and by this modulates root system architecture [48]. MYB73 is a positive regulator of auxin signaling [99,100]. *CAMTA1* has cell type specificity in expression pattern, which changes in development and in response to auxin transport inhibition are reminiscent of those demonstrated by the auxin sensor [101,102]. Transcriptome analyses of the *camta1* knockdown mutant reveal among 63 upregulated genes 17 associated with auxin signaling. Furthermore, both plants with repression of CAMTA1 protein activity and *camta1* knockdown mutant are hyper-responsive to auxin compared to wild type. Along with involvement in auxin signaling through regulation of its intermediates CAMTA1 participates in regulation of auxin transport and homeostasis and responds to various stresses. *HB13* and its paralogs redundantly control vein patterning in *A. thaliana*, likely via modulation of auxin homeostasis in the leaf blade [103].

USes, DSes and UAs also control auxin response. A number of AUX/IAA genes are repressed in plants, which overexpress the US *BMY2/BAM8* [104]. TCP20 enhances auxin conjugation by direct activation of *GH3.3* [105]. HB40 alters auxin distribution by deregulation of auxin transport [106].

DS RAP2.12 suppresses auxin-driven hypoxic root bending and hypoxia induced downregulation of PIN2 auxin transporter [107]. RAP2.12 abolished auxin upregulation of *LBD18* expression and ChIP–qPCR detected that RAP2.12 did this by binding to *LBD18* promoter [108]. Nevertheless, the co-immunoprecipitated fraction did not contain the RAP2.12 binding *cis*-element. This explains why this binding was not detected by DAP-seq. Yeast two-hybrid and BiFC assays demonstrated that RAP2.12 interacts with ARF7 and the Mediator subunit MED25 [108]. It provides the additional ARF7/MED25-RAP2.12-LBD18 link to our TFRN. In the root apical meristem of *crf10* mutant, *ARF7* had a significant increase in the expression level and expansion of the expression domain, whereas *CRF10* overexpression decreases *ARF7* expression [109]. UA LBD18 participates in the auxin control of lateral root initiation and development [25,110]. It was shown before that auxin inhibits DEL2 post-translationally [111]. Here we demonstrate that auxin upregulates *DEL2* transcriptionally promoting DEL2 upregulation of multiple BPs (Table S14).

For some of these TFs the key role in auxin regulated processes was also shown. *CAMTA1* loss-of-function mutation decreases chlorophyll content under drought conditions [112]. KUA1 homologue in alfalfa upregulates lignin biosynthesis in response to osmotic stress [113]. HB5, MYB70 and MYB73 are also involved in regulation of lignin biosynthesis [5,48,114,115,116]. In *A. thaliana*, overexpression of *HB13* increases chlorophyll content [117].

### 3.3. For Several TFRN TFs the Key Role in Auxin Regulated Processes Have Been Previously Shown

For some of TFRN TFs the regulation of auxin controlled processes was demonstrated. bZIP16 and bZIP53 were supposed to regulate chlorophyll biosynthesis in mesophyll cells [118]. *bZIP53* overexpressing plants have significantly reduced chlorophyll level compared to wild type [119]. bZIP16 is known as a transcription repressor of light activated genes [120]. Both genes also participate in regulation of ABA signaling [120,121]. bZIP16 is a primary transcriptional repressor of ABA action, which acts directly on several ABA-responsive genes and indirectly on some positive regulators of ABA signaling [120]. AT1G74840 is also involved in ABA signaling [122].

In wheat, overexpression of *CBF3* homolog increases chlorophyll content under drought and salt stresses [123]. In carrot, overexpression of *CBF3/DREB1a* homologue leads to an increase in lignin content and activity of enzymes for lignin biosynthesis [124]. *GBF3* is a hub in the reconstructed network. GLKs (GOLDEN2-LIKE) TFs provide root greening and are inhibited by auxin [53]. The capacity of GLKs to facilitate chlorophyll biosynthesis is strongly lost in the *A. thaliana gbf1/2/3* triple loss-of-function mutants, demonstrating the important role of GBF TFs in this BP [125]. GBF3 was shown the high tier TF in the TF hierarchy within the ABA response network [4].

HB21 increases ABA response by activation of ABA biosynthesis [126]. Here we show that HB21 also increases ABA level via activating *BG1*, which implements ABA deconjugation.

FAR-RED ELONGATED HYPOCOTYL 3 (FHY3) activates chlorophyll biosynthesis and it was demonstrated that FHY3 can do it by direct binding to the *HEME B1* promoter [127]. DAs *TGA4* [128] and *EPR1* [129] are also FHY3 directly activated targets and may be parts of additional pathways in FHY3 upregulation of chlorophyll biosynthesis.

For some TFs, indirect relation to lignin biosynthesis was shown. *BOP1* (*BLADE-ON-PETIOLE 1*) and *BOP2* overexpression upregulates lignin biosynthesis enzymes [130,131]. BOPs lack a DNA-binding domain and interact with TGACG-motif binding (TGA) basic Leu zipper (bZIP) transcription factors for recruitment to DNA. TGA4 is among essential BOP cofactors in regulation of development [132]. MYB73, ERF27, AT1G1900 and TGA5 were found as regulators of lignin biosynthesis, but their targets in this process identified by yeast one-hybrid assay [5] differ from those found by us. Probably this relates to complex regulation of lignin biosynthesis and its dependence on growth conditions.

In seedlings, auxin inhibits chlorophyll biosynthesis by direct ARF binding to promoters of *GUN5*, *ALB1/CHLD* and *HEMD* [54]. Here we show that activity of all these genes may be also decreased by auxin downregulation of their activators via tightly intertwined regulatory network and *ALB1/CHLD* is additionally the direct target of two USes, NAC47 and NAM (Figure 5).

Thus, in addition to 13 TFRN TFs known as regulators of auxin response, for 11 TFRN TFs there is evidence of their participation in auxin-driven processes. Thus, only for 25 (42%) TFRN TFs their role in auxin response and/or auxin-driven processes was previously recognized.

### 3.4. The Highly Connective TFRN Targets Play the Key Role in Auxin-Regulated Processes

For many dDEGs highly connected to the R-subnetwork, the key role in the BPs, which they tightly link to this subnetwork, was shown. For example, *PSEUDO-RESPONSE REGULATOR 5* (*PRR5*), which in circadian rhythm is activated by ten DAs, is the direct regulator of expression timing for key TFs in clock-output pathways [133]. *LOW PSII ACCUMULATION 3* (*LPA3*) is essential for photosystem II assembly [134] and is most tightly linked to R-subnetwork from dDEGs enriched in this BP. Reactive oxygen species are mainly generated in mitochondria, chloroplasts and peroxisomes in response to various abiotic stresses [135]. Glutathione peroxidases (GPX) protect plants against the oxidative stress. In the *A. thaliana* GPX family, GLUTATHIONE PEROXIDASE 6 (GPX6) is specifically localized in the mitochondria [136] and has the most pronounced differential expression under high light and cold stresses [137]. FindTFnet marks *GPX6* as the most connective gene between response to oxidative stress and the DA–DA part of the R-subnetwork (ten links). Among seven genes closely linking (nine links) response to water deprivation to the DA–DA part of the R-subnetwork, the key role in the process was shown for two ones. Overexpression of *BRO1-LIKE DOMAIN-CONTAINING PROTEIN* (*BRO1*) provides robust tolerance to drought [138], whereas *CONSERVED PEPTIDE UPSTREAM OPEN READING FRAME 46* (*CPuORF46*) increases in response to water limitation to regulate translation of any downstream *ORFs* [139]. *PAS/LOV DOMAIN PROTEIN* (*PLP*) has nine direct relations to the DAs and is the most DA connective protein in six following BPs: responses to light intensity, water deprivation, salt and oxygen-containing compound, cellular and organic substance catabolism. For three of these BPs, the key *PLP* role has been already demonstrated. PLP splicing variants play a critical role in a signature feature of high light acclimation, an increase in the ascorbate level, and dominate among blue light receptors during this process [140]. Also *PLP* transcript level drastically increases in response to salt or dehydration stresses [141]. *COLD REGULATED PROTEIN 27* (*COR27*) is a key gene in blue light and low temperature control of flowering [142,143]. *COR27* has 13 direct relations to the DAs and is the most DA connective gene in three responses (to ABA, blue light and cold). Thus, auxin down regulated activators before auxin treatment serve for maintenance of biological processes by fine tuning their key genes.

AUTOPHAGY 8 (ATG8) proteins play a central role in autophagy functioning and are used as reliable autophagosome markers [144,145]. In *A. thaliana*, among nine members of the ATG8 family, *ATG8e* is highest in roots [146]. We found that among autophagy genes downregulated by auxin, *ATG8e* is the most connected (nine links; Table S15) to the DA–DA part of the R-subnetwork. From them, five links to bZIP3, bZIP68, GBF3, TGA4 and TGA9 were shown previously by yeast one-hybrid assay using *ATG8e* 400 base pair promoter [147]. *TGA9* overexpression activated autophagy and transcriptionally up-regulated *ATG8e* expression via TGA9 protein binding to the *ATG8e* promoter.

The function of *AT5G15190* suppressed by the most number of USes (four TFs) in seven BPs (ABA signaling, catabolism of organic substances, responses to chitin, wounding, salt stress and water deprivation) is not yet studied, but nevertheless *AT5G15190* was mentioned among universal stress responsive genes reacting to heat, cold, salinity and drought [148]. *Pathogen-induced Cysteine-rich trans Membrane protein 7* (*PCM7*) is the most US suppressed gene in five BPs (ABA signaling, cellular catabolism, defense response to bacterium, responses to osmotic stress and water deprivation). *PCM7* is activated in response to various pathogens or their immune elicitors and connects these responses to photomorphogenesis [149]. PCM7 also protects plants against heat and UV stress [150]. *PUTATIVE INCREASED SNS1* (*INS1*) most suppressed in response to oxidative stress, wounding and light was previously shown as the constitutive stress-responsive gene upregulated by seven different stresses [151].

Six genes are targets of five UAs and among them there are *FASCIATA2* (*FAS2*) encoding one of three subunits of the H3-H4 histone chaperone complex CHROMATIN ASSEMBLY FACTOR 1, which deficiency leads to disorganized apical meristems [152]. The vital importance of several genes directly controlled by both DSes and four UAs was shown. Among them, there are *PIN1*, one of the key players in auxin distribution [153], *TRANSLOCASE OF THE INNER MEMBRANE 9* (*TIM9*) encoding a mitochondrial membrane protein, whose loss causes mitochondrial dysfunction resulted in embryo lethality [154] and *ORGANELLE TRANSCRIPT PROCESSING DEFECT 43 (OTP43)* gene, which mutants acquire undetectable mitochondrial Complex I and developmental defects [155].

### 3.5. Auxin-Regulated TFRN Implements a Trade-Off between Plant Development and Response to Environmental Cues

Auxin regulation of ribosome biogenesis was shown previously and was proposed as one of the tools for auxin control of developmental processes [156,157,158,159]. Phenotypes of mutants with loss of function of genes involved in ribosome biogenesis very often resemble auxin defects [160]. We have not found any previously published papers on relation of TFs from A-subnetwork to ribosome biogenesis and this certainly needs further research, but ribosome biogenesis is definitely related to growth patterning [161] and all UAs as the main part of the A subnetwork play the key roles in developmental transitions. DEL2 inhibits repressors of cell division [111]. *ERF4* upregulates cell size by promoting endopolyploidy and is specifically expressed in cells undergoing expansion [162]. *ERF11* elevates growth under both optimal and stress conditions [163,164]. MYB3R1 is a transcriptional activator of G2/M genes and promotes cytokinesis [165]. CRF10 and its direct target genes form the regulon with high activity in initials of various cell types that indicates its role in cell fate reprogramming [1]. LBD18 activates lateral root initiation and development [110,166].

The DSes communicate developmental patterning and stresses. ERF15 was determined as one of three key regulators in Casparian strip development [1] and mediator of plant defense responses [121]. The other DS RAP2.12 activates transcription of stress responsive genes [167] and meantime under hypoxia condition, inhibits *WOX5* transcription in the root quiescent center [108] and prevents shoot regeneration in calli [168].

Among USes, there are both key players in growth patterning and intermediaries between it and stress responses. BMY2 controls shoot growth and development [102]. TCP20 coordinates cell divisions and growth [169]. NAM regulates development and degeneration of outer integuments and by this way embryogenesis [170], and its loss of function reduce ethylene inhibition of lateral root development [171]. bZIP53 activates genes responsible for seed maturation [172] and reprograms metabolic pathways in response to salt and starvation stresses [119,173]. HB40 modulates cell division [106], reduces cell growth [174] and is induced by high temperature and osmotic stresses [175]. bZIP16 promotes seed germination and hypocotyl elongation [116] and its binding activity is redox dependent [176]. NAC47 is responsive to both water and drought stresses [177] and upon waterlogging stimulates local cell expansion in hyponastic leaf growth by increasing ethylene biosynthesis via direct upregulation of *ACC OXIDASE 5* (*ACO5*) [178].

All DAs participate in stress responses and most of them participate in control of balance between stress response and growth. bZIP68, the trigger from the first tier in R-subnetwork, is the stress sensor reacting to abiotic and biotic stresses by reducing its accumulation in the nucleus and increasing in cytoplasm [179]. Stresses disturb redox homeostasis, which leads to accumulation of reactive oxygen species [180]. This oxidative stress is sensed by bZIP68 [179]. Under favorable conditions, bZIP68 inhibits expression of stress tolerance genes and maintains expression of growth-related genes, whereas bZIP68 inactivation by moving to cytoplasm promotes stress tolerance but prevents growth. TGA4 and TGA9 are also involved in both redox signaling and regulation of growth and development [132,181,182,183]. Another major trigger from the first tier EPR1/RVE7 increases in response to warm temperatures and enhances the hypocotyl growth [184]. HB21 is negatively associated with genes of abiotic stresses, whereas HB21 repression causes severe defects in plant architecture and flower development and reduces plant height [185]. *HB13* and its paralogs redundantly control vein patterning in *A. thaliana*, likely via modulation of auxin homeostasis in the leaf blade [103]. AT1G19000, CBF3, HB13 are important in cold response [117,186] and BEH2 in various stresses [187]. *GBF3* overexpression provides tolerance to both bacterial infection and drought and their combination, which is very frequent in field conditions [188]. TGA4, TGA5 and TGA9 have the central roles in defense response [189]. VERNALIZATION1 (VRN1) also links external cues to development. VRN1 responds to long-term exposure to cold stress (vernalization) by the silencing of *Flowering Locus C* (*FLC*) via changing its chromatin structure [190]. This promotes transition to flowering. VRN1 also controls other aspects of *A. thaliana* development [191]. bZIP50 stimulates flowering and is involved in drought and disease responses [192]. BPC1 and LCL1/RVE4, DAs, for which links to the TFRN were not found also play important roles in both development and stress responses [193,194,195].

*CAMTA1* is a part of a gene network linking auxin signaling and adaptive responses to changes in environment [101,102]. We can hypothesize that the role described for CAMTA1 as link between auxin controlled development and phenotypic plasticity in adaptation to environment may be suggested for the whole R-subnetwork.

Thus, our results demonstrate a promising outlook of using a new approach for computational inference of TFRNs, including the ones triggered by other signaling pathways. The reconstructed TFRNs can successively guide the design of experimental studies investigating transcriptional regulation of biological processes. Since the current version of FindTFnet uses a library of *A. thaliana* TF binding profiles limited to about 30% of *A. thaliana* TFs, the future directions include the expansion of the collection of *A. thaliana* peak sets, and accumulation of representative collections of TF binding profiles for other species.

## 4. Methods

### 4.1. Publicly Available Datasets Used in the Study

For the reconstruction of auxin-regulated TFRN, we used publicly available microarray data for *A. thaliana* roots (GSE42896) either exposed to auxin (samples GSM1053033, GSM1053034, and GSM1053035) or not exposed to the hormone (samples GSM1053036, GSM1053037, and GSM1053038) [34]. The seedlings were germinated in liquid MS medium supplemented with 10 μM naphthylphthalamic acid (NPA, the auxin transport inhibitor) under continuous light conditions (photosynthetically active radiation). Three days after germination, the seedlings were transferred to MS medium with 10 µM 1-naphthalene-acetic acid (NAA, a synthetic auxin) for 6 h. The list of DEGs between the samples exposed to auxin and the untreated control samples collected before auxin treatment was extracted from [35]. To complement DAP-seq-derived ARF family TFs’ binding loci (see below), we used publicly available ChIP-seq data for ARF3/ETTIN (ETT) [14] and ARF6 [13]. The ARF6 peak set was downloaded in BED format from GTRD database (PEAKS042831) (https://gtrd.biouml.org/, accessed on 23 March 2023, [196]). Raw ARF3/ETT ChIP-seq data (PRJEB19862) were retrieved from NCBI Sequence Read Archive (SRA) (https://www.ncbi.nlm.nih.gov/sra/, accessed on 25 June 2024). ChIP-seq-derived ARF5, ARF7 and ARF10 target genes were taken from [15]. The genome sequence of *A. thaliana* (TAIR 10) was downloaded from Ensembl Plants (https://plants.ensembl.org/index.html, release 52, accessed on 25 June 2024).

### 4.2. Implementation of FindTFnet as a Part of CisCross Web-Server

We implemented a three-step procedure for the reconstruction of TFRNs based on the analysis of DEG lists (Figure 1A) as CisCross-FindTFnet module within the CisCross web-server, which we have developed previously [32]. For the enrichment analysis of transcription factor binding loci in promoter regions of uDEGs and dDEGs, CisCross-FindTFnet employs previously implemented CisCross-Main function [32]. To map TF binding loci, CisCross-Main recruits one of three in-built collections of *A. thaliana* TF peak sets [32] each being a result of alternative processing of raw DAP-seq data [16]. Next, to classify the enriched TFs as transcriptional activators or suppressors, we developed CisCross-FindRegulatedTF function. Of the enriched TFs, it extracts the ones, which can be assigned to one of the four types (DA, DS, US or UA) based on a certain set of rules (Figure 1). Namely, TF is assigned to DA type if its binding peaks are enriched in the promoters of dDEGs, and it is encoded by a dDEG as well. TF is assigned to DS type if its binding peaks are enriched in the promoters of uDEGs, while it is encoded by a dDEG. TF is assigned to US type if its binding peaks are enriched in the promoters of dDEGs, while it is encoded by a uDEG. TF is assigned to UA type if its binding peaks are enriched in the promoters of uDEGs, and it is encoded by a uDEG as well. We also identified such types of TFs as DR/UR and NTR, which we did not take in further analysis. TF is assigned to DR/UR type if its binding peaks are enriched in the promoters of both dDEGs and uDEGs, while it is encoded by a dDEGs/uDEGs. TF is assigned to NTR (non-transcriptionally regulated) type if its binding peaks are enriched in the promoters of dDEGs or/and uDEGs, but it is not encoded by any DEG. Finally, to select potential “TF regulator–TF target” pairs among all detected DAs, DSes, USes and UAs, we developed the CisCross-TF-targets function (Figure 1A). For each DA, DS, US or UA (regulator), it identifies DA/DS/US/UA-coding genes (targets), which possess the regulator binding peaks within their 5′ regulatory regions. The link type (activation or inhibition) between TF regulator and TF target is assigned according to the predicted function of TF regulator (activator or suppressor). TFRN visualization is implemented with the visNetwork R package (https://visjs.org/, https://github.com/visjs/vis-network, accessed on 15 November 2022). FindTFnet is available as a part of CisCross web-server at https://plamorph.sysbio.ru/ciscross/FindTFnet_index.html.

### 4.3. Reconstruction of Auxin-Induced TFRN

To reconstruct auxin-induced transcriptional cascade, we ran the new CisCross-FindTFnet module of CisCross web-server using a pair of uDEG/dDEG lists as input data (https://plamorph.sysbio.ru/ciscross/FindTFnet_index.html, accessed on 10 February 2023). Araport 11 release of *A. thaliana* genome annotation was selected to map transcription start sites (TSS) of genes. The length of the 5′ regulatory regions of interest was set as 1000 bp upstream TSS. To map TF binding loci in the genome, we used the CisCross-MACS2 DAP-seq collection of TFs peak sets, which consisted of 608 peak sets for 404 TFs [32]. During enrichment analysis of TF binding peaks in 5′ regulatory regions of DEGs, FDR was controlled at 0.001 with the Benjamini–Hochberg procedure. During reconstruction of “TF regulator–TF target” pairs, the peak sets corresponding to leaf gDNA possessing epigenetic DNA modifications (“col” data) and the ones corresponding to leaf gDNA with methylcytosines eliminated due to PCR amplification (“colamp” data) were processed separately. FindTFnet is not capable of univocally characterizing enriched TFs encoded by the stimulus-insensitive genes as activators or suppressors, therefore, we filtered them out from further analysis.

### 4.4. Functional Annotation of DEGs and Establishing a Relationship between TFRN and BPs

GO enrichment analysis of u/dDEG lists separately was done using DAVID [197] with FDR kept below 0.001. For each enriched BP, in its gene list we searched for DAP-seq peaks for DAs and USes in promoters of dDEGs and for DSes and UAs in promoters of uDEGs using CisCross_TF-targets function, which was implemented as a separate web page in CisCross web-server (https://plamorph.sysbio.ru/ciscross/TF_index.html, accessed on 10 March 2023). For this search, we used the CisCross-MACS2 DAP-seq collection of TF peak sets [32] and 1000 bp-long regulatory regions upstream TSS.

### 4.5. Raw ARF3/ETT ChIP-seq Data Analysis

To map ARF3/ETTIN binding loci in *A. thaliana* genome, we processed publicly available ChIP-seq data (PRJEB19862) [14] using a standard pipeline. Namely, we applied FastQC v0.12.1 (http://www.bioinformatics.babraham.ac.uk/projects/fastqc, accessed on 25 June 2024) for the reads quality control, Fastp v0.23.4 [198] for the raw data preprocessing, Bowtie2 v2.5.4 [199] for the reads alignment to the *A. thaliana* reference genome, and MACS3 v3.0.1 [200] for peak calling. The wild-type sample with no auxin treatment (ERS1589529) was used as a control when running MACS3 callpeak function for the transgenic *pETT:ETT-GFP ett-3* samples. The biological replicates were processed separately.

### 4.6. DNA Motif Search

To extract the nucleotide sequences of 1000 bp-long regulatory regions upstream of *AT1G19000*, *GBF3*, *ERF11*, *ERF15*, and *bZIP16* TSSes from the *A. thaliana* genome, we used Bedtools getfasta function [201]. For the motif search, we used two sets of Position Weight Matrices (PWMs) for bZIP16, bZIP3, bZIP68, bZIP53, GBF3, BMY2, TGA4, TGA5, TGA9, BEH2, ERF4, ERF15, ERF11, CRF10, and LBD18. The first set was available in the JASPAR database [202], and represented a result of de novo motif search from the corresponding DAP-seq peaks [16]. The second set was previously retrieved from the same reprocessed DAP-seq data (CisCross-MACS2 collection of peak sets) [32]. Motif search was performed with universalmotif (https://bioconductor.org/packages/universalmotif/, accessed on 25 June 2024). PWM thresholds were set according to the log-odds algorithm [203].

### 4.7. Accession Numbers, Full Names and Other Names for Genes Encoding TFs from the Auxin Induced TFRN

DAs. *BEH2* (AT4G36780, *BES1/BZR1 HOMOLOG 2*), *BPC1* (AT2G01930, *BASIC PENTACYSTEINE 1*), *VRN1* (AT3G18990, *REDUCED VERNALIZATION RESPONSE 1*, *REPRODUCTIVE MERISTEM 39/REM39*), *CAMTA1* (AT5G09410, *CALMODULIN-BINDING TRANSCRIPTION ACTIVATOR 1*); AP2/ERF (APETALA2/ETHYLENE RESPONSIVE FACTOR) family, *ERF27* (AT1G12630, *ERF027*), *CBF3* (AT4G25480, *C-REPEAT BINDING FACTOR 3*, *DEHYDRATION RESPONSE ELEMENT B1A/DREB1A*); bZIP (BASIC LEUCINE-ZIPPER) family, *bZIP3* (AT5G15830), *bZIP50* (AT1G77920, *TGACG SEQUENCE-SPECIFIC BINDING PROTEIN 7/TGA7*), *bZIP68* (AT1G32150), *GBF3* (AT2G46270, *G-BOX BINDING FACTOR 3*), *TGA4* (AT5G10030, *OCS ELEMENT BINDING FACTOR 4/OBF4*), *TGA5* (AT5G06960, *bZIP26*, *OCS ELEMENT BINDING FACTOR 5/OBF5*), *TGA9* (AT1G08320, *bZIP21*); HD-ZIP (HD-ZIP, HOMEODOMAIN LEUCINE ZIPPER) family, *HB13* (AT1G69780, *HOMEOBOX 13*, *ATHB13*), *HB21* (AT2G18550, *ATHB21*), *HB5* (AT5G65310); MYB (V-MYB AVIAN MYELOBLASTOSIS VIRAL ONCOGENE HOMOLOG) family, *EPR1* (AT1G18330, *EARLY-PHYTOCHROME-RESPONSIVE1*, *REVEILLE 7/RVE7*), *KUA1* (AT5G47390, *KUODA1*, *MYB HYPOCOTYL ELONGATION-RELATED/MYBH*), *LCL1* (AT5G02840, *LHY/CCA1-LIKE 1*, *REVEILLE 4/RVE4*), *MYB70* (AT2G23290), *MYB73* (AT4G37260). USes. *BMY2* (AT5G45300, *BETA-AMYLASE 2*, *BETA-AMYLASE 8/BAM8*), *TCP20* (AT3G27010, *TEOSINTE BRANCHED 1*, *CYCLOIDEA*, *PCF (TCP)-DOMAIN FAMILY PROTEIN 20*); bZIP (BASIC LEUCINE-ZIPPER) family, *bZIP16* (AT2G35530), *bZIP53* (AT3G62420); HD-ZIP (HD-ZIP, HOMEODOMAIN LEUCINE ZIPPER) family, *HB18* (AT1G70920), *HB40* (AT4G36740); NAC (NAM—NO APICAL MERISTEM, ATAF-*ARABIDOPSIS THALIANA* ACTIVATING FACTOR, CUC-CUP-SHAPED COTYLEDON) family, *NAM* (AT1G52880, *NAC018*, *NAC-REGULATED SEED MORPHOLOGY 2/NARS2*), *NAC47* (AT3G04070, *NAC047*, *SPEEDY HYPONASTIC GROWTH/SHYG*). DSes. AP2/ERF (APETALA2/ETHYLENE RESPONSIVE FACTOR) family, *ERF15* (AT2G31230), *RAP2.12* (AT1G53910, *RELATED TO AP2.12*, *ERF74*). UAs. *LBD18* (AT2G45420, *LOB DOMAIN 18*, *ASYMMETRIC LEAVES2 LIKE 20/ASL20*), *DEL2* (AT5G14960, *DP-E2F-LIKE 2*, *E2FD*, *E2F TRANSCRIPTION FACTOR-LIKE E2FD 1/E2L1*); AP2/ERF (APETALA2/ETHYLENE RESPONSIVE FACTOR) family, *ERF4* (AT3G15210, *RELATED TO AP2.5/RAP2.5*), *ERF11* (AT1G28370), *CRF10* (AT1G68550, *CYTOKININ RESPONSE FACTOR 10*); MYB (V-MYB AVIAN MYELOBLASTOSIS VIRAL ONCOGENE HOMOLOG) family, *MYB3R1* (AT4G32730, *MYB TF WITH 3 MYB REPEATS 1*).

Accession numbers of genes encoding enzymes of chlorophyll and lignin biosynthesis, components of ABA signaling pathway and ribosome biogenesis are given in Tables S9A, S10A, S11A and S14A, respectively.

## Figures and Tables

**Table 1:** Literature-based verification of the functional types of auxin response regulators predicted with FindTFnet.

Regulator Type/Predicted Function	No. of TFs	No. of Predictions with No Experimental Data Available *	No. of Predictions Contradictory to Experimental Data *	No. of Predictions Supported by Experimental Data/of Them with Known Dual-Function *
DA/activator	23	2	7	14/5
DS/suppressor	2	0	2	0/0
UA/activator	6	0	0	6/4
US/suppressor	8	1	5	2/1
Total	39	3 (8%)	14 (36%)	22 (56%)/10 (26%)

* The detailed list of TFs and references are in Table S3.

## Data Availability

FindTFnet is available at https://plamorph.sysbio.ru/ciscross/FindTFnet_index.html. All data supporting the findings of this study are available within the paper and its Supplementary Materials.

## Supplementary Materials

The following supporting information can be downloaded at: https://www.mdpi.com/article/10.3390/plants13141905/s1, Table S1: The list of differentially expressed genes (DEGs); Table S2: FindTFnet output and its processing; Table S3: Verification of the functional types of TF regulators predicted with FindTFnet; Table S4: Functional annotation of DEGs: David output on Biological Processes; Table S5: Links of Biological Processes to the Downregulated Activators (DAs); Table S6: Links of Biological Processes to the Upregulated Suppressors (USes); Table S7: The most DA and US connective genes in the BPs enriched in auxin downregulated DEGs; Table S8: Auxin downregulates chlorophyll biosynthesis by upregulating suppressors that repress both genes encoding enzymes for this process and TFs—activators of these genes; Table S9: Auxin downregulates lignin biosynthesis by upregulating suppressors that repress both genes encoding enzymes for this process and TFs—activators of these genes; Table S10: Auxin downregulates ABA signaling by upregulating suppressors that repress both genes encoding components for this process and TFs—activators of these genes; Table S11: Links of BPs to the Downregulated Suppressor (DSes); Table S12: Links of BPs to the Upregulated Activators (UAs); Table S13: BP activation by UAs during auxin treatment; Table S14: Auxin upregulates ribosome biogenesis by downregulating TFs suppressing genes encoding components for this process in norm and upregulating TFs activators of these genes; Table S15: DAs having DAP-seq peaks in the ATG8E 1000 bp promoter; Supplementary Figure: The robust part of the auxin-regulated TFRN in *A. thaliana* roots.
