# Supplementary material for: Computational Reconstruction of the Transcription Factor Regulatory Network Induced by Auxin in Arabidopsis thaliana L
Source: Plants (Basel). 2024 Jul 10;13(14):1905. doi: 10.3390/plants13141905 (PMC11280061; doi:10.3390/plants13141905)
Supplement: Supplementary file 1 [file plants-13-01905-s001.zip › plants-3062661-supplementary.pdf]

1 Supplementary Figures

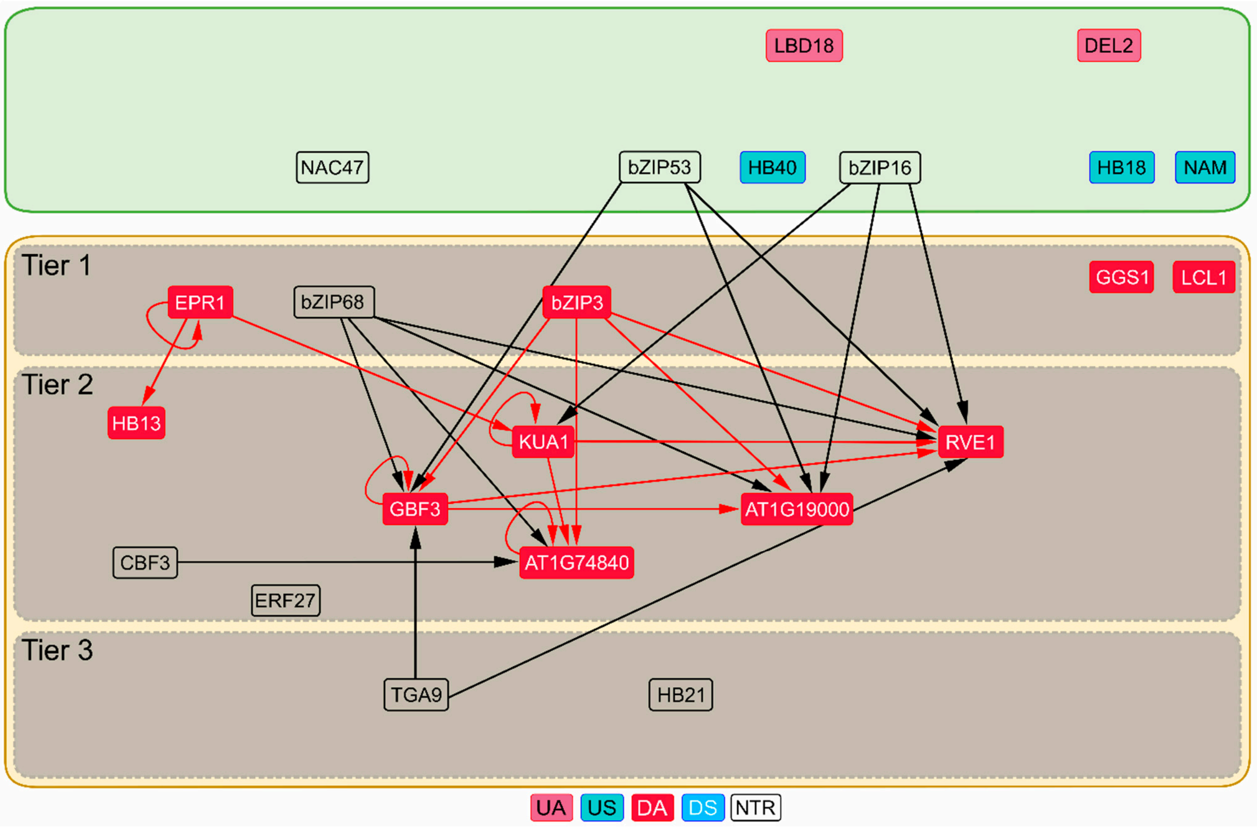

Supplementary Figure. The robust part of the auxin-regulated TFRN in *A. thaliana* roots. The network was predicted with FindTFnet using the list of DEGs with the fold change of at least 2 (i.e. with the log fold change of at least 1). A repressed subnetwork (R-subnetwork) is highlighted with a yellow box, an activated subnetwork (A-subnetwork) is highlighted with a green box. Gray boxes denote three tiers of R-subnetwork. UA, upregulated activator; US, up-regulated suppressor; DA, downregulated activator; DS, downregulated suppressor. NTR (non-transcriptionally regulated), TF-coding gene which is not DEG but the encoded TF's binding loci are enriched in the regulatory regions of DEGs.
